# 

**Published:** 1869-08

**Authors:** 


### INDEX.

### A.

Absorption of Nutriment Injected Subcutaneously ... 21
Abstract of Proceedings of the Buffalo Medical Association, 1, 41, 87, 121, 179, [301, 401
Action of Mercury Podophyline and Taraxacum on the Biliary Secretions ... 307
Address before the Convention of Delegates from Medical Colleges. By Prof. D. W. Yandell, M.D., of Louisville, Ky. 361
Address betore the Graduating Class of the Medical Department of the University of Buffalo By Walter Clark, D.D. 322
Albany City Hospital ... 275
Albany Medical College ... 356
Al en. Interm rriage of Relations ... 319
Allen. Physical Culture in Amherst College ... 157
American Exch nge and Review ... 36
American Journal of Syphilography and Dermatology ... 235
American Ophthalmological Society, Transactions of, at Fourth and Fifth Annual Meetings ... 36
American Medical Associ tion, Transac- tions of ... 233
American Medical Association ... 311
" Association of Medical Editors ... 477
" Library of ... 32
" Twenty first Annual Convention of ... 372
" Proceedings of ... 391
" Official Report of, Meeting of ... 429
" Meeting of ... 352
American Ophthalmological and Otological Societies ... 278
Anaesthetics, Modus Operandi of ... 67
Animal Poisoning, a case of. By Z. C. McElroy, M.D., of Zanesville, Ohio ... 247
Animal Vaccination and Vacc inal Syphilis ... 229
Annual Address before the New York State Medical Society. By Prof. James P. White, M.D , President ... 281
Annual Report of the Surgeon Ge eral ... 238
Apoplexy, Meningeal, Death from. By James S. Bailey, M.D., of Albany, N.Y. 371
Apothecaries' Act ... 19
Archives of Ophthalmology and Otology 435
Arlt. Tre tment of Lachrymal Affections 119
Artificial Respiration : Dr. Benj. Howard's Direct Method of ... 26
Atlantic Monthly ... 79
Atlee, Dr. William L., Communication from ... 93
Aural Catarrh or Otites Media, By E M. Curtis, M. D- ... 480

### B.

Bailey. James S., Death from Meningeal Apoplexy ... 371
Baldwin, Andrew. Address before the American Medic l Association ... 159
Baltimore Medical Journal ... 235
Barnes Obstetric Operations, with Selections, by Benjamin Dawson, M. D. ... 477
Beard. Our Home Physician ... 37
Be nett Pr ctice of Medicine ... 358
Biliary Secretions : Action of Mercury, Podophyline and Taraxacum on ... 307
Bingham Hall; A Hospital for Insane, at Canandaigua, Report of ... 438
Biostatic Immunities of the Jewish Race in Europe ... 54
Bigelow. The Mechanism of Dislocation and of Fracture ot the Hip Reduction of Dislocation by the Flexion Method ... 155
Bones, Morbid Elongation of ... 141
Books and Pamphlets Received, 40, 80, [120. 160, 200, 240, 280, 320, 360, 400. 440, 480
Brain Softening, after Coal Gas Poisoning 267
Bristol, Dr. Moses, Death of: Action of Erie County Medical Society ... 150
Bromide of Potassium, Cutaneous Eruptions due to ... 268
Browne. Virchows Life of the Trichina. 36
Buffalo State Insane Asylum ... 399
" Clinical Teaching on the Subject of Insanity ... 421
Butler Hospital for Insane, at Providence, R. I., Report of ... 437

### C.

Carbolic Acid, Internal use of ... 96
Carbolic Acid, Effects of on the Human Economy, on Vegetable Parasites and diseases of the Skin ... 147
Carroll. Hygiene in its relations to Therapeutics ... 158
Diseases of the Skin ... 147
Case of Poisoning by Nicotina ... 20
Cataplasms. the Philosophy of ... 350
Chicago Medical Journal ... 160
Chambers on Indigestions, ... 478
Chloral ... 142
" An Antidote to ... 419
" Clinical Experience with ... 305
" Therapeutic Note on ... 413
" Transformation of Hydrate of Nitro Chloroform in the Human Organism ... 417
Chloroform: Influence of in Promoting Cutaneous Absorption ... 309
Chloroform, and Sir James Y. Simpson ... 262
" Sudden Death during the Operation of Ovariotomy, while the Patient was under the Influence of ... 234
Circular No. 2; Surgeon General's Office, on Excisions of the Head of the femur for Gunshot Injury ... 76
Clark, Walter, D.D., Address to the Graduating Class of the Medical Department of the University of Buffalo ... 322

Clinical Instruction in Insanity and Diseases of the Nervous System........ 238
Coal Gas Poisoning, Softening of Brain after........ 267
College of Physicians of Philadelphia, Transactions of........ 38
Colles' Fracture, New Treatment for........ 356
Commencement at the Buffalo Medical College........ 239
Commencement Exercises at the Buffalo Medical College: Names of Graduates 269
Cook, A. B. Joined Twins: their Obstetrical and Surgical Management........ 37
Correction........ 279
Cottage Hospitals........ 28
Communication from Prof. J. P. White, M. D., Containing a Letter from Dr. Atlee, of Philadelphia........ 65
" From Romaine J. Curtis, M.D........ 67
" From Dr. William L. Atlee, of Philadelphia........ 93
" From Prof. J. P. White, M.D........ 157
Completion of Vol. Nine, of Buffalo Medical and Surgical Journal........ 477
Compression of the Spinal Cord........ 103
Crepitant and Sub Crepitant Rale, Mechanism of........ 139
Criminal Abortion........ 114
Criticism on the Management of the case of the late Dr. March........ 235
Curtis, Romaine J., M.D., Communication........ 67
Criticism on Two Cases of Double Spontaneous Dislocation of the Lens........ 261
Cutaneous Absorption of Iodide of Potassium........ 309
Cutaneous Eruptions, due to Bromide of Potassium........ 268

**D.**

Death of Prof. Jas. Hadley, M.D. Resolutions of the Medical Profession 112
" Dr. J. J. Edmunds. Action of the Erie County Medical Society........ 70
" Dr. Joshua R. Lothrop. Resolutions of the Medical Staff of the Buffalo General Hospital 29
" Sir James Y. Simpson, M D........ 399
" Meeting of American Physicians at Washington; Addresses and Resolutions respecting the same........ 423
" Dr. Moses Bristol. Action of Erie County Medical Society 150
Death from Chloroform. Fatty Degeneration of the Heart. By J. F. Miner, M.D........ 215
Delegate to the National Convention, for the Revision of the Pharmacopœa........ 357
Determination of the Size of the Roots of Teeth previous to Extraction........ 58
Diagnosis of Tubercular Meningitis, by means of the Ophthalmascope........ 16
Disease, Influence of Nutriment on........ 143
Dispersion of Uterine Fibroids........ 110

**E.**

Eastman, Prof N. H., M.D., on the Action of Mercury........ 201
Eczema, Treatment of........ 311
" Scabie's and Hebra's Treatment of........ 20
Edinburgh's Part in the History of Anaesthesia........ 262
Edmunds, Dr. J. J., Death of. Action of Erie County Medical Society........ 70
Elbow, Dislocation of; New Method of Reduction........ 144
Electrical Apparatus, Kidder's........ 315
Electricity in Obstetrics........ 418
Electrolysis, Treatment of Malignant Tumors by........ 58
Elephantiasis Arabum........ 225
Emmet. Surgery of the Cervix in connection with certain Uterine Diseases. 319
Erichson. Science and Art of Surgery........ 118
Erie County Medical Society Dinner........ 239
Exposed Pulps........ 350

**F.**

Forceps........ 23
" Intra Uterine........ 146
Francis Dr. John W., Portrait of........ 277
Flint. Philosophy of Man........ 317
Fownes Chemistry........ 75

**G.**

Garretson. Diseases and Surgery of the Mouth, Jaws, and associate parts........ 117
Georgia State Medical Association........ 37
Good Health........ 37
Gray. Anatomy, Descriptive and Surgical........ 437
Gunn. Luxations of the Hip and Shoulder Joints, with the agents that oppose their reduction........ 199
Galezowski's Binocular Strabometer........ 25
Gay, C. C. F., M.D. Two Cases of Labor Complicated by the Presence of Uterine Tumors........ 41
" Sudden Death from supposed Heart Disease........ 181
Genesee County Medical Society, Annual Meeting of........ 230
Gibbs. O. C., M.D., of Frewsbury. Communication on Puerpual Eclampsia, etc 221

**H.**

Hadley, Prof. James, M.D., Death of. Meeting of the Medical Profession........ 112
Hamilton, G. W., M.D. Catarrh of the Uterus........ 368
Hamilton. Plastic Operations........ 277
Hammond. Sleep and its Derangements 315
Hare Lip, New Method of Using Needle in Operation for........ 226
Hay Fever caused by Vibriones........ 416
Heart Disease, Sudden Death from Supposed. By C. C. F. Gay, M.D........ 181
Hebra's Management of Scabies and Eczema........ 20
Hodge. Fœticide. or Criminal Abortion 159
Hospital Association, Ladies........ 429
Hospitals, Cottage........ 28
Homœopathy, Modern........ 20
Howard University, Catalogue of........ 32
Howard, v. Dr. Benjamin. Direct Method of Artificial Respiration........ 26
Human Heart, Work Done by........ 349
Huxley. Physical Basis of Life........ 318

**I.**

Illinois State Medical Society, Transactions of........ 359
Indiana State Medical Society, Transactions of........ 79
Iodide of Potassium, Absorption of by the Skin........ 309
Indirect Osteo-Plasty........ 22
Index to Vol. IX........ 481
Infants, Use of Starchy Food for........ 52
International Medical Congress of 1869........ 27
Intra Uterine Forceps........ 196
Iron, Puchloride of. Dangers of Injection of in Sanguineous Tumors........ 146
Items, Selections and Remarks. By W. W. Miner, A.B. 30, 71, 151, 193, 231, [273, 312, 353, 394, 426

K.

Kansas City College of Physicians and Surgeons ........ 198
Knapp. A Treatise on Intra Ocular Tumors ........ 236
Knee Joint, Wounds of. By J. F. Miner, M.D. ........ 333

L.

Lawrence. Hand Book of Ophthalmic Surgery ........ 157
Lawson. Diseases and Injuries of the Eye ........ 274
Lead Poisoning ........ 224
Lecture, Introductory to the Course on Materia Medicæ and Hygiene, delivered at the University of Buffalo, Nov. 28th, 1869. By Prof. Charles A. Lee, M.D ... 161
Lehlbach. Carbolic Acid, its Action and Uses ........ 158
Lens, Two Cases of Spontaneous Dislocation of ........ 223
Library of American Medical Association at Washington, D. C. ........ 32
Lothrop, Joshua R., M.D., Death of. Resolutions of Medical Staff of General Hospital ........ 29

M.

Massachusetts General Hospital, A Visit to ........ 218
McElroy, Z. C., M.D. A Case of Animal Poisoning, with Remarks upon the Modus Operandi of Organic Poisons ........ 247
Mechanism of the Crepitant and Sub-Crepitant Rale ........ 139
Medical and Surgical Cases, occurring in the Sisters' of Charity Hospital, in the service of Prof. Rochester, *v.* Prof. Eastman. Reported by W. W. Miner, A.B. ........ 81, 128
Medical Education of Women ........ 191
Medical Adviser ........ 235
Medical Journals and Periodicals ........ 238
Medical Progress. An oration delivered by A. N. Bell, M. D ........ 432
Medical Prizes ........ 191
Medical Profession in Rome, and the Papacy ........ 104
Medical Staffs of Hospitals, Rights of ... 227
Mercury, On the Action of. By Prof. N. H. Eastman, M.D., of Geneva Medical College ........ 209
Metropolitan Board of Health, Third Annual Report ........ 78
Medicinal Appliances on Swedish Railroads ........ 29
Medicines as Disturbing Agents ........ 148
Meigs. Practical Treatise on Diseases of Children ........ 357
Membrana Tympani, Complete Destruction of both. Application of Artificial Drum, with great benefit ........ 142
Michigan State Medical Society, Proceedings of ........ 36
Missouri State Medical Society, Transactions of ........ 438
Michigan University Journal ........
Miner, J. F., M.D. Rupture of the Perineum ........ 7
" A case of Morphine Poisoning, apparently relieved by Atropine ........ 134
" Death from Chloroform. Fatty Degeneration of the Heart ........ 215
" Wound of the Knee Joint ........ 333
Miner, W. W., A.B. Report of Medical and Surgical Cases occurring in the Sisters' of Charity Hospital ........ 77, 128
Modern Homœopathy ........ 20
Morphine Poisoning, apparently relieved by Atropia, a case of. By J. F. Miner, M.D. ........ 134
Modus Operandi of Anaesthetics ........ 57
Myers. Medical Electricity ........ 116

N.

Napheys. Modern Therapeutics ........ 432
" Physical Life of Woman ........ 319
Naval Staff Rank, Principles and History of ........ 198
National Convention for the Revision of the United States Pharmacopœia ........ 154
National Convention of Medical Colleges 229
National Medical Society of the District of Columbia ........ 229
" " Memorial to ........ 271
Naval Discipline ........ 107
Nebraska State Medical Society, Meeting of ........ 439
Nervous Disease, Ophthalmoscope in ... 147
Nervous System in Disease ........ 101
New York State Medical Society, Annual Meeting of ........ 276
" " Address before. By Prof. James P. White, M.D... 241
Nicotina, case of Poisoning by ........ 20
Niemeyer's Text Book of Practical Medicine ........ 47
Northampton State Lunatic Asylum, Report of ........ 439
Notice to the Public ........ 353
Noyes. Glaucoma ........ 359
Nutriment, Absorption of, Subcutaneously Injected ........ 21

O.

Obstetrics, Electricity in ........ 418
Odling. A Course of Practical Chemistry for Medical Students ........ 159
Ohio State Medical Society, Transactions of ........ 198
Ophthalmia Sympathetic, Enucliation of the Eye in. By E. N. B. Smith, M.D. 90
Ophthalmoscope in Nervous Disease ........ 147
Oration, by J. M. Toner, M.D., before the Medic l Society of the district of Columbia ........ 39
Oregon Medical and Surgical Reporter ... 166
Osteo-Plasty, Indirect ........ 22
Ovariotomy ........ 25
Oxyuris Ermicularies ........ 316

P.

Physicians Problems ........ 479
Personal Beauty. How to cultivate and preserve it ........ 479
Papacy and the Medical Profession in Rome ........ 104
Pennsylvania State Medical Society, Transactions of ........ 115
Photographs of Distinguished Foreign Physicians, Surgeons and Specialists .. 29
Phthisis, Relation of Pulmonary Hemorrhage to ........ 350
Physicians' Hand Book ........ 197
" Visiting List ........ 119
Physiological Effects of Muscular Exercise ........ 470
Pysical Life of Women ........ 478
Prognosis in Hepatic Abscess ........ 474
Passage of the White Blood Corpuscles through the capillary vessels ........ 474
Pneumonia, Treatment of ........ 348
" " ........ 56
Potter, H. H., M D., of Geneva, Death of 166
Politzer. The Membrana Tympani, in Health and Disease ........ 78

Prize Essays 489
Professional Success 262
Pure Stimulants 30

Q.

Quarrels, Professional and Personal 352

R.

Reasons why the Profession should labor to diffuse a general knowledge of Medical Science among the people 11
Reviews. Soelberg Wells, on Diseases of the Eye 34
Rheumatism 26
Riley. Compend of Materia Medicæ 198
Rockwell. Electricity as a means of Diagnosis 199
Rupture of the Uterus 106
Rupture of the Perineum. By J. F. Miner, M.D. 7

S.

Sawyer. Obstetric Aphorisms 278
Sayre. Club Foot 38
Scabies and Eczema, Hebra's Management of 20
Scarlet Fever, Treatment of 418
Scientific and Literary Publications 398
Scientific and Literary Publications 237
Schoeppe, Dr. Paul, the case of 187
" " Intercession for 197
Simpson, Sir James Y., Death of 399
" Address and Resolutions of a Meeting of American Physicians respecting 423
Skin, Diseases of, Effects of Carbolic Acid on 147
Smith, E. N. B., M.D. Enucliation of the Eye in Sympathetic Ophthalmia 90
Smithsonian Institution, Annual Report of 317
Smith. On Wasting Diseases of Children. 278
Snelling. Nocturnal Enuresis and Incontinuence of Urine 439
" Relaxation of the Pelvic Symphyses during Pregnancy and Parturition 436
Spinal Cord Compression of 103
Spinal Irritation 345
Squibb, E. R., M.D. Notes on the Phenols from Coal Tar, and on Rhubarb 35[illegible]
Starchy Food for Infants 52
State Insane Asylum, Meeting of Commissioners on Location of 197
Strabometer, Galezowski's Binocular 25
" " " 113
Sudden Death from supposed Heart Disease. By C. C. F. Gay, M.D. 181
Suit for Mal-practice 314
Surgeons against Juries and Shysters 259
Syphilis, on the influence of, in production of Tubercle 419

T.

Tanner. Manual of Clinical Medicine and Physical Diagnosis 438
Tape Worm, Treatment of 226
Taunton State Lunatic Asylum, Report of 439
Teachers' Convention at Washington 398
Teeth, Roots of, Determination of Size of previous to Extraction 58
Texas State Medical Society, Proceedings of 591
Treatment of Paralysis 575
The Nation 77
The Probe 39
Thomas Chronic Inversion of the Uterus 318
Tubercle, Inoculation of 307
" Influence of Syphilis on the Production of 419
Tumors, Malignant, Treatment of by Electrolysis 58
Tumors, Sanguineous; Dangers of Injection of Perchoride of Iron into 146
Tympanitis, Intestinal Puncture in 19
Tyson. Cell Doctrine 430

U.

Utica State Medical Society, Twenty-seventh Annual Report of 433

V.

Vogel. Practical Treatise on Diseases of Children 277

W.

Williams. Diseases of the Eye 432
United States Sanitary Memoirs 480
Uterine Fibroids, Dispersion of 110
Uterine Tumors, Two cases of Labor complicated by the presence of. By C. C. F. Gay. M.D. 41
Uterus, Catarrh of. By G. W. Hamilton, M.D., of Orangeville, Ohio 368
Uterus, Rupture of 106
Vaccinal Syphilis and Animal Vaccination 229
Vectis 22
Vision of Animals 473
Vegetable Parasites, Effects of Carbolic Acid on 147
What is Pneumo Typhus ? 18
What is Senile Pneumonia ? 18
What is Typhoid Pneumonia ? 17
White, Prof. J. P., MD. Communication containing a Letter from W. Atlee, of Philadelphia 65
" " Communication 157
" " Annual Address before the New York State Medical Society 241
" " Inaugural Address before the New York State Medical Society 281
Women, Medical Education of 191
Yandell, Prof. D. W. Address before the Convention of Delegates from Medical Colleges 361

### Books and Pamphlets Received.

A Text-Book of Practical Medicine, with particular reference to Physiology and Pathological Anatomy, by Dr. Felix von Niemeyer, Prof. of Pathology and Therapeutic, Director of the Medical Clinic of the University of Tubingen, translated from the seventh German edition, by special permission of the author; by George H. Humphreys, M. D., one of the Physicians to the Bureau of Medical and Surgical Relief at Bellevue Hospital for the out-door poor, Fellow of the New York Academy of Medicine, etc., and Charles E. Hackley, M. D., one of the Physicians to the New York Hospital, one of the Surgeons to the New York Eye and Ear Infirmary; Fellow of the New York Academy of Medicine, etc.

Volume I and II New York, D. Appleton & Co., 1869. Received through, and for sale by Breed & Lent, Buffalo.

Electricity in its Relations to Practical Medicine. By Dr. Moritz Meyer, Royal Counselor of Health, etc. Translated from the third German edition, with notes and additions, by William A. Hammond, M. D., Professor of Diseases of the Mind and Nervous System, and Clinical Medicine, in Bellevue Hospital Medical College; Vice-President of the Academy of Medical Science, National Institute of Letters, Arts and Sciences; late Surgeon General United States Army, etc. New York, D. Appleton & Co., 1869. Received through and for sale by Breed & Lent, Buffalo.

Third Annual Report of the Metropolitan Board of Health of the State of New York. Albany, Charles Van Benthuysen & Son, 1868.

Circular No. 2, War Department, Surgeon General's Office, Washington, January 2d, 1869. Report on Excisions of the Head of the Femur for Gunshot Injury.

The Membrana, Tympani in Health and Disease. Illustrated by Twenty-four Chromo-Lithographs. Clinical contribution to the Diagnosis and treatment of Diseases of the Ear, with supplement, by Dr. Adam Palitzer, of the University of Vienna, translated by A. Mathewson, M. D., and H. G. Newton, M. D., Assistant Surgeons of the Brooklyn Eye and Ear Hospitals. New York, Wm. Wood & Co. 1869. Recieved through and for sale by Breed & Lent, Buffalo.

Announcements.—Eleventh Annual Announcement of the Chicago Medical College Session of 1869–70.

St Louis College of Physicians and Surgeons, Session 1869-70.

Annual Announcement and Catalogue of the Missouri Medical College, Session 1869–70.

Annual Announcement and Catalogue of the Detroit Medical College, March Session, 1870.

Fifth Annual Announcement of the St. Louis College of Pharmacy, Session 1869–70.

Annual Address Delivered by M. O. Baldwin, M. D., before the American Medical Association at New Orleans, May, 1869.

On External Perineal Urethrotomy, or an Improved method of External Division of the Urethra in Perinæo for the Relief of Obstinate Stricture, with remarks on the preparatory and after-treatment, by J. W. Gouley, M. D., Professor of Clinical Surgery, and of Genito-Urinary Diseases, in the University of New York, Surgeon to Bellevue Hospital, etc. New York, D. Appleton & Co., 1869.

Fœticide, or Criminal Abortion. A lecture introductory to the course of Obstetrics and Diseases of Women and Children. University of Pennsylvania, Session 1839–40, by Hugh Hodge, M. D. New York, D. Appleton & Co., 1869.

Transactions of the Indiana State Medical Society, Indianapolis, May 19th and 20th, 1869.

The System of Public Instruction, State and City of New York.

The Illustrated Annual of Phrenology and Physiognomy, by S. R. Wells, Editor of the Phrenological Journal and Life Illustrated. New York, 1869

Catalogue of Books published by Hurd & Houghton. New York H. O. Houghton & Co., Cambridge, Mas.

